# Accurate quantification of lipid species affected by isobaric overlap in Fourier-transform mass spectrometry

**DOI:** 10.1016/j.jlr.2021.100050

**Published:** 2021-02-16

**Authors:** Marcus Höring, Christer S. Ejsing, Sabrina Krautbauer, Verena M. Ertl, Ralph Burkhardt, Gerhard Liebisch

**Affiliations:** 1 Institute of Clinical Chemistry and Laboratory Medicine, Regensburg University Hospital, Regensburg, Germany; 2 Department of Biochemistry and Molecular Biology, Villum Center for Bioanalytical Sciences, University of Southern Denmark, Odense, Denmark; 3 Cell Biology and Biophysics Unit, European Molecular Biology Laboratory, Heidelberg, Germany

**Keywords:** lipidomics, mass spectrometry, lipids, phospholipids, sphingolipids, triglycerides, isotope correction, data processing, Fourier-transform mass spectrometry, peak interference, AGC, automated gain control, CE, cholesteryl ester, Cer, ceramide, DB, double bond, DG, diglyceride, FIA, flow injection analysis, FTMS, Fourier-transform mass spectrometry, FWHM, full-width at half maximum, HRMS, high-resolution MS, LOQ, limit of quantification, LPC, lysophosphatidylcholine, LPE, lysophosphatidylethanolamine, PC, phosphatidylcholine, RIA, relative isotopic abundance, SM, sphingomyelin, TG, triglyceride

## Abstract

Lipidomics data require consideration of ions with near-identical masses, which comprises among others the Type-II isotopic overlap. This overlap occurs in series of lipid species differing only by number of double bonds (DBs) mainly because of the natural abundance of ^13^C-atoms. High-resolution mass spectrometry, such as Fourier-transform mass spectrometry (FTMS), is capable of resolving Type-II overlap depending on mass resolving power. In this work, we evaluated FTMS quantification accuracy of lipid species affected by Type-II overlap. Spike experiments with lipid species pairs of various lipid classes were analyzed by flow injection analysis-FTMS. Accuracy of quantification was evaluated without and with Type-II correction (using relative isotope abundance) as well as utilizing the first isotopic peak (M+1). Isobaric peaks, which were sufficiently resolved, were most accurate without Type-II correction. In cases of partially resolved peaks, we observed peak interference causing distortions in mass and intensity, which is a well-described phenomenon in FTMS. Concentrations of respective species were more accurate when calculated from M+1. Moreover, some minor species, affected by considerable Type-II overlap, could only be quantified by M+1. Unexpectedly, even completely unresolved peaks were substantially overcorrected by Type-II correction because of peak interference. The described method was validated including intraday and interday precisions for human serum and fibroblast samples. Taken together, our results show that accurate quantification of lipid species by FTMS requires resolution-depended data analysis.

Lipidomics is an emerging discipline in life sciences providing structural details and quantities for hundreds of lipid molecules in biological specimen including tissues, body fluids, cells, or subcellular compartments (1, 2, 3, 4, 5). A major issue of lipidomic analysis is the substantial isotopic overlap of lipid species differing only by the number of double bonds (DBs). Particularly, the natural abundance of ^13^C-atoms (about 1.1%) contributes significantly to the second isotopic peak resulting in intensities of >10% related to the monoisotopic peak of a typical glycerophospholipid. The overlap of the monoisotopic peak (M^DB=i^+0) with the second isotopic peak of a species of the same lipid class and number of C-atoms but with one additional DB (M^DB=i+1^+2) is termed Type-II isotopic effect (6). In low resolution mass spectrometry, this effect is frequently corrected by algorithms based on calculated isotope patterns in both shotgun (6, 7) and LC-based lipidomic methods (8). In contrast, the application of high-resolution MS (HRMS) allows the resolution of a Type-II overlap caused by incorporation of two ^13^C-atoms with a mass difference of 8.94 mDa of M^i+1^+2 and M^i^+0 (9). Additionally, accurate quantification of lipid species in both, low resolution mass spectrometry and HRMS, requires consideration of decreasing abundance of the monoisotopic peak with increasing C-atom numbers (Type-I isotopic effect) (6).

In recent years, HRMS lipidomic approaches increasingly apply Fourier-transform mass spectrometry (FTMS) (9, 10, 11, 12, 13). However, distortions in mass and intensity of ions with near-identical masses is a known phenomenon for FTMS analysis (14, 15, 16). These distortions can be related to peak coalescence of the ion clouds, which become phase-locked producing a single peak instead of two (14, 17). Occurrence of peak coalescence is related to increasing number of ions. Furthermore, several distortions can be related to FT signal processing (16). These issues were also observed in FTMS-based lipidomics (9, 18) and can be linked to a distorted relative isotopic abundance (RIA) (15, 16, 19, 20). In the following, these distortions will be summarized and termed as peak interference. The application of the first isotopic peak M+1, which is less affected by Type-II overlap than the monoisotopic peak, was suggested to identify and quantify such lipid species (18).

In this work, we evaluated the accuracy of quantification of lipid species affected by Type-II overlap by flow injection analysis (FIA)-FTMS. Concentration derived from three different data processing strategies, i.e., without and with Type-II correction and application of M+1 were evaluated. Finally, a workflow depending on mass resolving power was proposed to minimize peak interference effects as far as possible.

## Materials and Methods

### Reagents and lipid standards

Chloroform and 2-propanol were purchased from Roth (Karlsruhe, Germany) and methanol from Merck (Darmstadt, Germany). All solvents were HPLC grade. Ammonium formate and cholesteryl ester (CE) standards were purchased from Sigma-Aldrich (Taufkirchen, Germany). Triglyceride (TG) and diglyceride (DG) standards were purchased from Larodan (Solna, Sweden) and dissolved in 2,2,4-trimethylpentane/2-propanol (3:1 v/v). Phosphatidylcholine (PC), ceramide (Cer), sphingomyelin (SM), lysophosphatidylcholine (LPC), and lysophosphatidylethanolamine (LPE) standards were purchased from Avanti Polar Lipids (Alabaster, AL) and dissolved in chloroform.

### Biological samples

Human serum samples were collected from residual patient material after clinical routine diagnostics. Murine liver samples were pooled residuals of previous studies. Primary human skin fibroblasts were cultured in Dulbecco's Modified Eagle Medium supplemented with L-glutamine, nonessential amino acids, and 10% fetal calf serum at 5% CO_2_ in a humidified incubator at 37°C as described previously (21).

### Standard mixtures

Two standard mixtures were prepared. The mixture “M0” contained the following lipid species: LPE 18:0 (0.018 pmol/μl, concentration in the infusate), LPC 18:0 (0.18 pmol/μl), DG 36:0 (0.18 pmol/μl), CE 18:0 (0.18 pmol/μl), SM 36:1;2 (0.18 pmol/μl), Cer 42:1;2 (0.018 pmol/μl), and TG 54:0 (0.18 pmol/μl). The mixture “M1” was composed of the corresponding species of the same lipid class with exactly one additional DB: LPE 18:1 (0.018 pmol/μl), LPC 18:1 (0.18 pmol/μl), DG 36:1 (0.18 pmol/μl), CE 18:1 (0.18 pmol/μl), SM 36:2;2 (0.18 pmol/μl), Cer 42:2;2 (0.018 pmol/μl), and TG 54:1 (0.18 pmol/μl). A volume of 25 μl of both mixtures subjected to lipid extraction, corresponding to 0.018 pmol/μl (0.1 nmol added to lipid extraction) LPE and Cer, and 0.18 pmol/μl (1 nmol added to lipid extraction) LPC, DG, CE, SM, and TG was defined as ratio M1/M0 = 1:1. Samples were analyzed with a constant amount of the M0 mixture and variable amounts of the M1 mixture. Concentrations of the stock solutions were adjusted by FTMS quantification using the respective internal standards. The internal standard mixture used for quantification was composed of CE 17:0 (0.7 pmol/μl), Cer 32:1;2 (0.005 pmol/μl), DG 28:0 (0.11 pmol/μl), LPC 13:0 (0.15 pmol/μl), LPE 13:0 (0.011 pmol/μl), PC 28:0 (0.23 pmol/μl), SM 30:1;2 (0.007 pmol/μl), and TG 51:0 (0.26 pmol/μl). The concentration of the infusate was calculated by dividing the amount added to the extraction in nmol by a factor of 5.598 derived from dilution during sample preparation.

### Lipid extraction

Samples were spiked with internal standards before lipid extraction (solvent of standards was removed by vacuum centrifugation). A serum amount of 10 μl, cell homogenate containing 100 μg of protein, or tissue homogenates containing a wet weight of 2 mg were subjected to extraction. The samples were extracted according to the procedure described by Bligh and Dyer (22) with a total chloroform volume of 2 ml. An amount of 500 μl of the separated chloroform phase was transferred into a sample vial by a pipetting robot (Tecan Genesis RSP 150) and vacuum dried. The residues were dissolved in 1.4 ml of 7.5 mM ammonium formate in chloroform/methanol/2-propanol (1:2:4 v/v/v).

### Direct flow injection high-resolution MS

Lipid quantification was performed by direct flow injection on a hybrid quadrupole-Orbitrap mass spectrometer QExactive (Thermo Fisher Scientific, Bremen, Germany) equipped with a heated electrospray ionization source and a standard-sized Orbitrap. The ion source was operated using the following settings: spray voltage of 3.5 kV, S-lens RF level 50, capillary temperature of 250°C, aux gas heater temperature of 100°C, and settings of 15 for sheath gas and 5 for aux gas. Enhanced Fourier-transform was applied for signal processing (23). All data were acquired in profile mode. Amounts of 50 μl of the reconstituted sample extracts were injected by a PAL autosampler (CTC Analytics, Zwingen, Switzerland) equipped with an UltiMate 3000 isocratic pump (Thermo Fisher Scientific, Waltham, MA). Chloroform/methanol/2-propanol (1:2:4 v/v/v) was delivered at an initial flow rate of 100 μl/min until 0.25 min followed by 10 μl/min for 2.5 min and a wash out with 300 μl/min for 0.5 min. Positive ion mode FTMS data (TG, DG and CE as ammoniated adducts) were recorded in *m/z* range 500–1,000 for 1 min. Negative ion mode FTMS data were recorded in *m/z* range 400–650 for LPE ([M–H]^−^) and LPC ([M+HCOO]^−^) and *m/z* range 520–960 for Cer, SM, and PC quantification (as [M+HCOO]^−^). All experiments used a maximum injection time of 200 ms, an automated gain control (AGC) of 1 × 10^6^, three microscans, and a target resolution of 140,000 (at *m/z* 200). Changes in resolution and AGC are indicated in the corresponding figures.

### Direct infusion nanoelectrospray ionization high-resolution MS

Samples were infused with the robotic nanoflow ion source TriVersa NanoMate (Advion Biosciences, Ithaca, NY) connected to an Orbitrap Fusion Tribrid equipped with a high-field Orbitrap analyzer (Thermo Fisher Scientific, San Jose, CA) as described previously (9). Full scan FTMS data were acquired in positive ion mode for 2 min in scan range *m/z* 470–1,030 using profile mode, a max injection time of 100 ms, AGC of 1 × 10^5^, three microscans, and a target resolution setting of 480,000 and 120,000. Negative ion mode was performed in a second injection in scan range *m/z* 360–675 and *m/z* 530–1,080 for 2 min each using the parameters as described before.

### Data processing and quantification

All FTMS spectra were processed using the ALEX software (24). The export of individual spectral peak lists to averaged peak lists of specific FTMS scan ranges was supported by the MSFileReader. ALEX includes lock-mass correction, peak assignment with *m/z*-tolerance set to ±0.0045 for intensity picking. The extracted data were exported to Microsoft Excel 2016 and further processed by self-programmed Macros. For the isotope Type-II effect (25), three different data processing strategies were compared. “Uncorrected” data were not corrected for the Type-II effect. “Corrected” values were corrected for the Type-II effect by a stepwise correction based on theoretical isotope distributions (7, 26, 27). As suggested by Wang *et al.* (18), the intensity of the first isotopic peak (M+1) was used for quantification (“M+1 quantification”). All data processing strategies include correction of abundance of peak abundance, i.e., Type-I effect (6). Calculation of RIA was performed with eMASS (28). The quantification was performed with nonendogenous internal standards by multiplication of the spiked IS amount with the analyte-to-IS ratio of the intensities after data processing.

## Results

### Mass spectra of Type-II overlapping lipid species

**Fig. 1:**
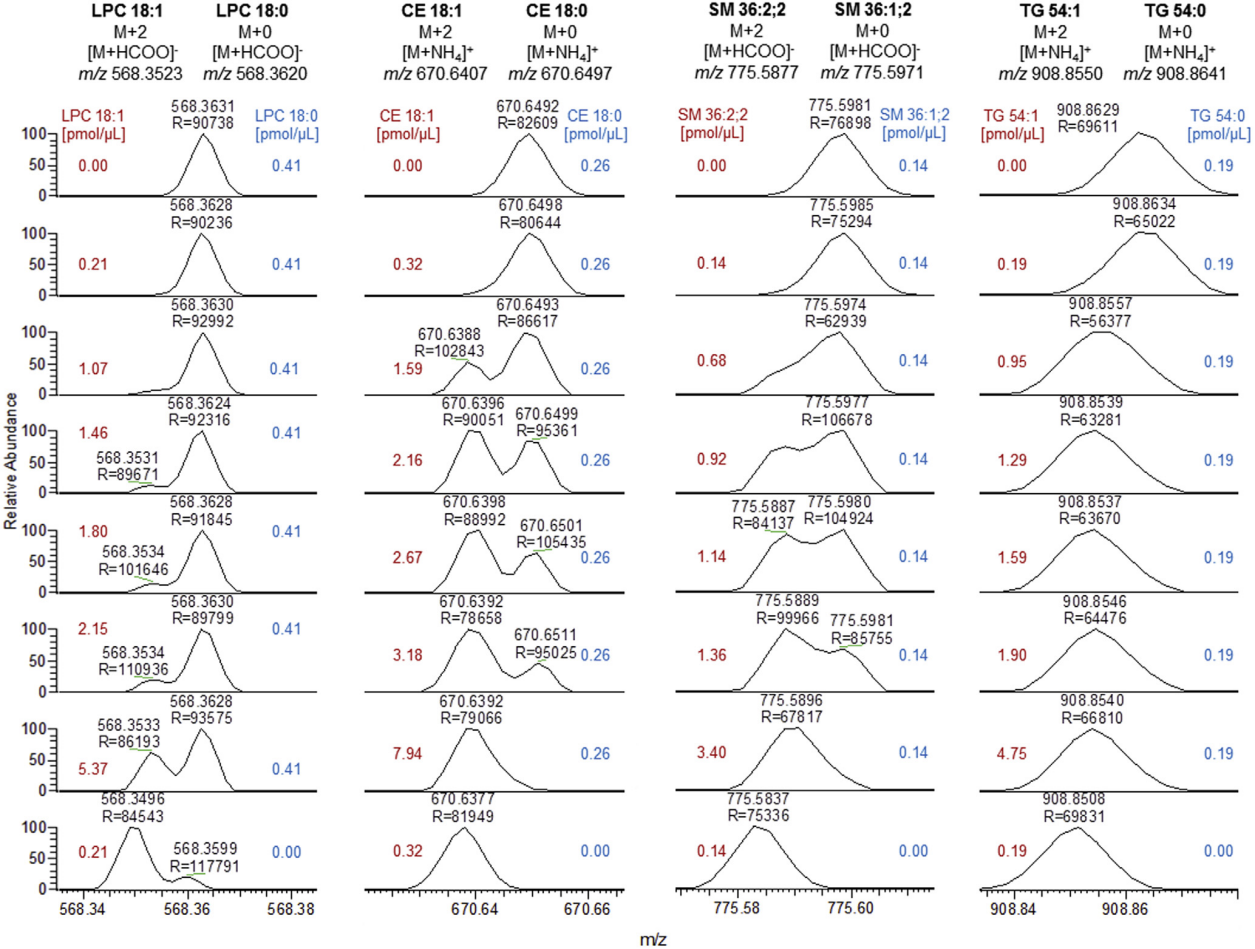
Mass spectra showing the DB overlap of selected lipid species pairs. The figure illustrates changes of peak shape and *m/z* shifts with increasing abundance of M^i+1^+2. Of note, spectra were not corrected for *m/z* offset. The in-vial concentration of the species M^i+1^ and M^i^ is indicated in red and blue, respectively. Data were recorded on a QExactive Orbitrap at a resolution setting of 140,000 (at *m/z* 200). DB, double bond.

To evaluate Type-II overlap in lipidomics data, a variety of synthetic lipid standard pairs, merely differing in one DB, was analyzed by FIA (29). Lipid standard pairs were analyzed with a constant concentration of species M^i^ and increasing concentrations of species M^i+1^ on a QExactive Orbitrap using FTMS at a mass resolution setting of 140,000 [full-width at half maximum (FWHM) at *m/z* 200]. In Orbitrap mass analyzers, mass resolution R is inversely proportional to the square root of *m/z* (30). Accordingly, resolution of isobaric peaks of M^i+1^+2 and M^i^+0 decreased with increasing *m*/*z*, resulting in completely unresolved peaks as shown for TG at *m/z* 909 (Fig. 1). Notably, the intensity ratio of M^i+1^+2 and M^i^+0 affected the peak resolution. Furthermore, partial peak resolution may be lost if the intensity of one peak significantly exceeds the other. Mass accuracy was also affected for ions with near-identical masses. Thus, as expected and demonstrated for TG, the detected *m/z* of the unresolved overlap resembled the peak of the more abundant ion.

### Quantification strategies

**Fig. 2:**
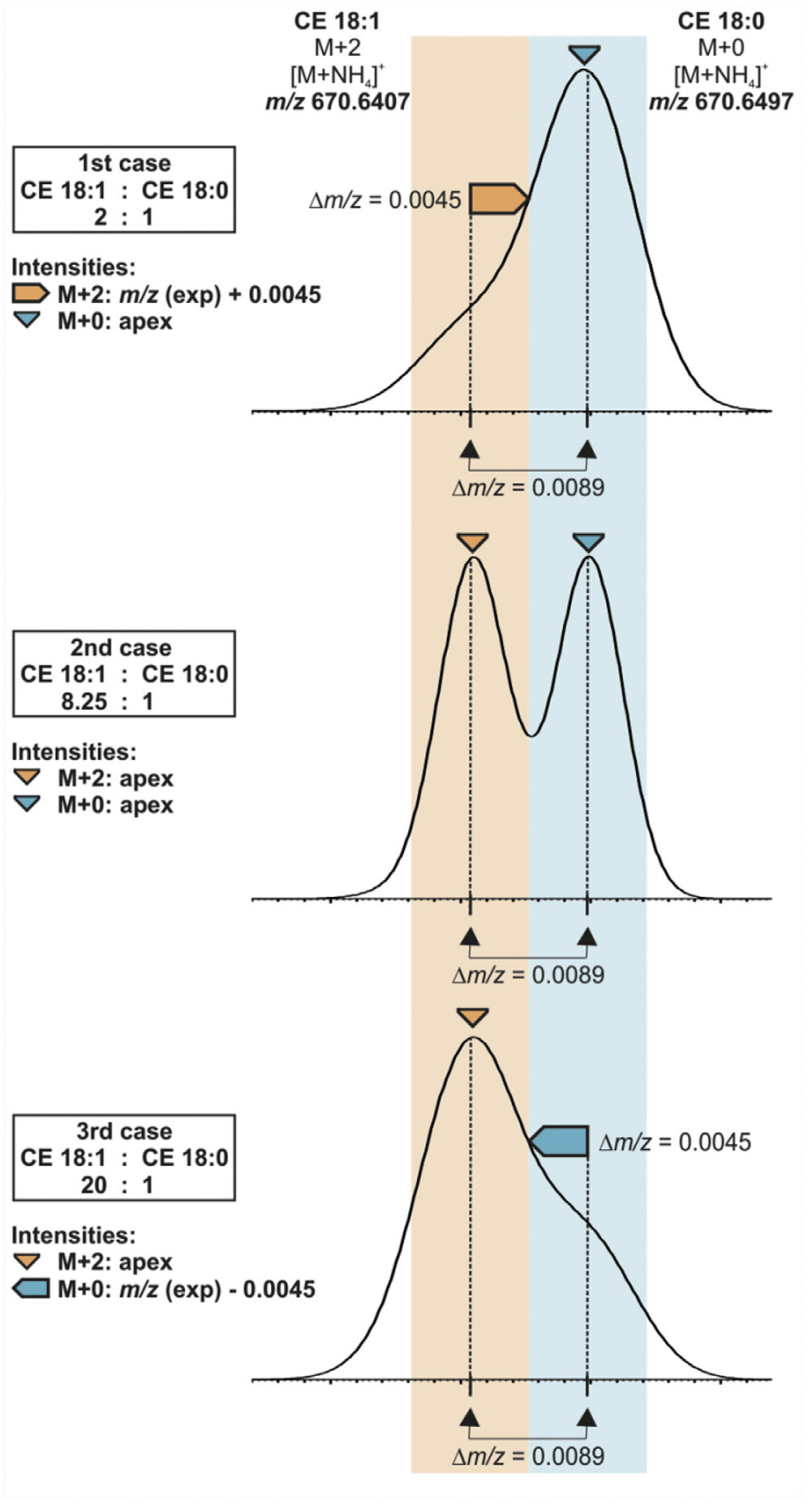
Peak picking and configurations. Peak configurations (also termed cases) and picking of intensity illustrated for the second isotope (M+2) of CE 18:1 and monoisotopic peak (M+0) of CE 18:0. Peak intensities are picked using ALEX software either at the apex (triangles) or at peak flank at *m/z* (expected) ±0.0045. The *m/z*-tolerance of ±0.0045 for intensity picking of M+2 and M+0 is displayed in orange and blue, respectively. The simulated spike ratio of the monoisotopic species is indicated in the figure legend. Simulations were performed using QualBrowser software (Thermo Fisher) and a target resolution of R = 100,000. CE, cholesteryl ester.

In the next step, we investigated lipid species quantification considering the impact of peak interference. Peak profiles were analyzed by ALEX software (24), which picks intensities as illustrated in Fig. 2. Depending on peak resolution and apex position of the isobaric peaks, we distinguished three peak configurations (also termed cases). Quantification was achieved using nonendogenous internal standards for each lipid class. To investigate peak interference, quantification was performed either without further correction of peak intensities (uncorrected), after Type-II correction using a stepwise correction based on theoretical isotope patterns (corrected) (7) or using the first isotopic peak M+1 (M+1 quantification) (18). Finally, peak intensities were subjected to Type-I isotope correction (6).

### Accuracy of quantification for lipid standards

**Fig. 3:**
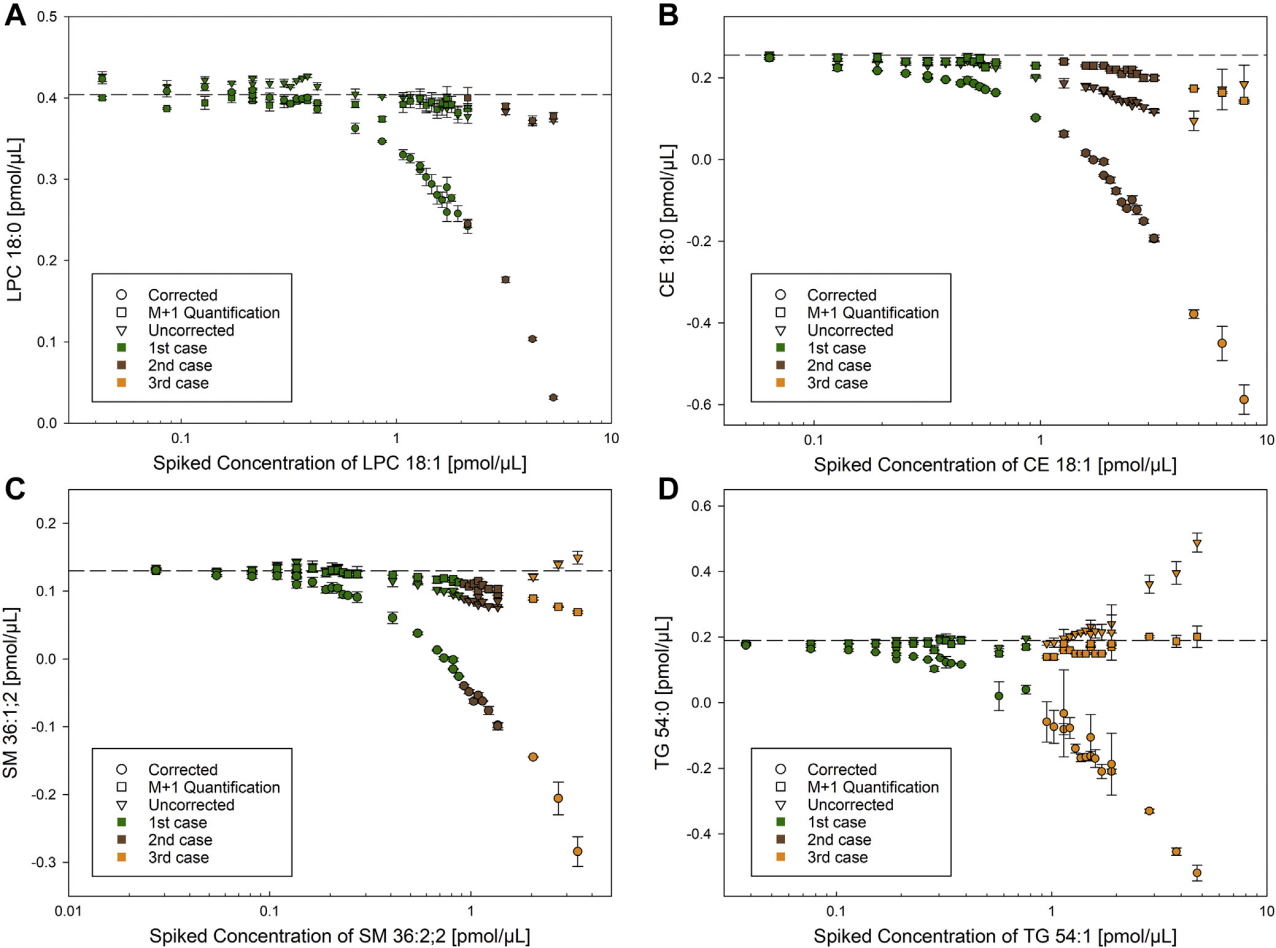
Comparison of data processing strategies. Quantification of (A) LPC 18:0 *m/z* 568.362, (B) CE 18:0 *m/z* 670.6497, (C) SM 36:1;2 *m/z* 775.5971 and (D) TG 54:0 *m/z* 908.8641 at increasing concentrations of the corresponding species with one additional double bond at resolution setting of 140,000 (at *m/z* 200). The figure displays concentrations calculated without (uncorrected, triangles), with (corrected, circles) Type-II correction and by the use of M+1 (squares). The dashed line indicates the spiked amount of the more saturated species. The color code describes the case of peak configuration as defined in Fig. 2 and is only related to the monoisotopic peak (M^i+1^+2 and M^i^+0) but not M+1 (M^i+1^+3 and M^i^+1). Each point represents the average of n = 3 technical replicates ± SD. CE, cholesteryl ester; LPC, lysophosphatidylcholine; SM, sphingomyelin; TG, triglyceride.

In the following, the accuracy of quantification was evaluated by comparison of measured and the known spiked concentration. As expected, quantification of LPC 18:0 (*m/z* 568.362; Fig. 3A) and LPE 18:0 (*m/z* 480.3096; supplemental Fig. S1A) did not require any correction for accurate quantification because of sufficient resolution of M^i+1^+2 and M^i^+0. In contrast, species with higher *m/z* revealed a pronounced deviation of the “uncorrected” quantification at higher spike amounts of species M^i+1^ even though the isobaric peaks were still partially resolved. For peaks with a configuration 1 or 2, M+1 quantification significantly improved the accuracy of quantification (CE 18:0 in Fig. 3B, SM 36:1; 2 in Fig. 3C; DG 36:0 *m/z* 642.6031 and Cer 42:1;2 *m/z* 694.6355 in supplemental Fig. S1B, C). Unexpectedly, Type-II correction of TG 54:0 (*m/z* 908.8641) led to a substantial overcorrection (Fig. 3D) despite M^i+1^+2 and M^i^+0 being completely unresolved at all spike ratios (Fig. 1). Here, the uncorrected quantification was accurate even for several case three configurations, except for highest spike levels of TG 54:1. Similar concentrations were achieved for the M+1 quantification, and here, even for the highest spike levels, target concentrations were accurate. Of note, the reported peak configuration was determined for the monoisotopic peak. For case 3 configurations, the intensity is picked from the peak flank and not the apex and should be considered with great care. For M+1 of TG 54:0, intensities were still picked at the apex except for the three samples with the highest spike concentration of TG 54:1.

### Evaluation of peak interference

**Fig. 4:**
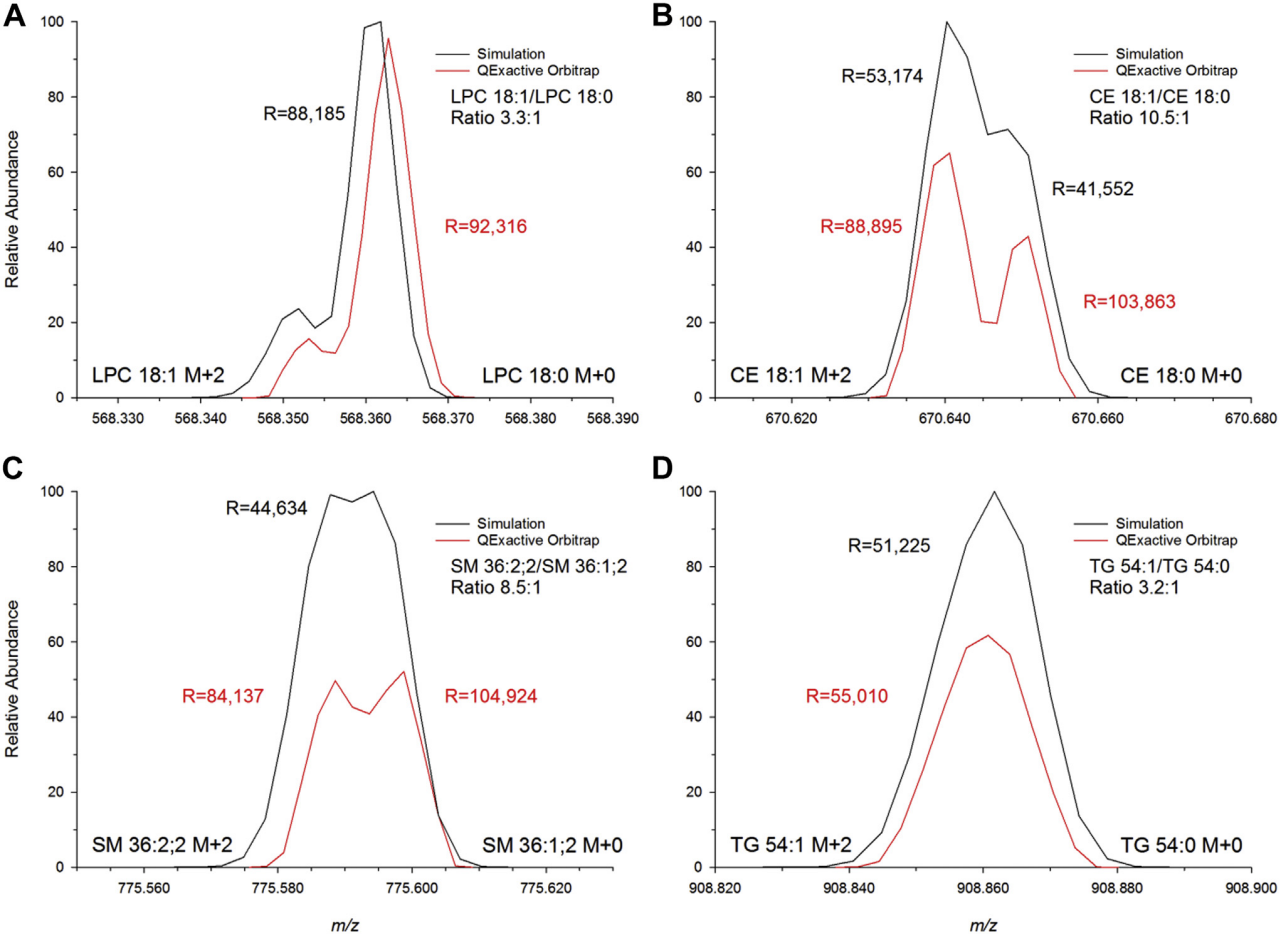
Loss of intensity of ions with near-identical masses. Comparison of measured and simulated profiles of the isobaric overlap of M^i+1^+2 and M^i^+0 of (A) LPC 18:1 and LPC 18:0, (B) CE 18:1 and CE 18:0, (C) SM 36:2;2 and SM 36:1;2, and (D) TG 54:1 and TG 54:0 for the indicated spike ratios. Mass spectra were acquired on a QExactive Orbitrap with a resolution setting of 140,000 (at *m/z* 200). Simulations were performed using QualBrowser software. Abundance of measured and simulated profiles was referenced by the monoisotopic peak of the more unsaturated species. In vial concentrations were: (A) LPC 18:1 (1.37 pmol/μl) and LPC 18:0 (0.41 pmol/μl), (B) CE 18:1 (2.67 pmol/μl) and CE 18:0 (0.26 pmol/μl), (C) SM 36:2;2 (1.14 pmol/μl) and SM 36:1;2 (0.14 pmol/μl), (D) TG 54:1 (0.57 pmol/μl) and TG 54:0 (0.19 pmol/μl). CE, cholesteryl ester; LPC, lysophosphatidylcholine; SM, sphingomyelin; TG, triglyceride.

Next, we investigated measured peak profiles and compared them with simulations. The comparison revealed distorted profiles for CE 18:0 (*m/z* 670.6497), SM 36:2;2 (*m/z* 775.5971) and to a lesser extent even for TG 54:0 (*m/z* 908.8641) but not for LPC 18:0 (*m/z* 568.3620) (Fig. 4).

**Fig. 5:**
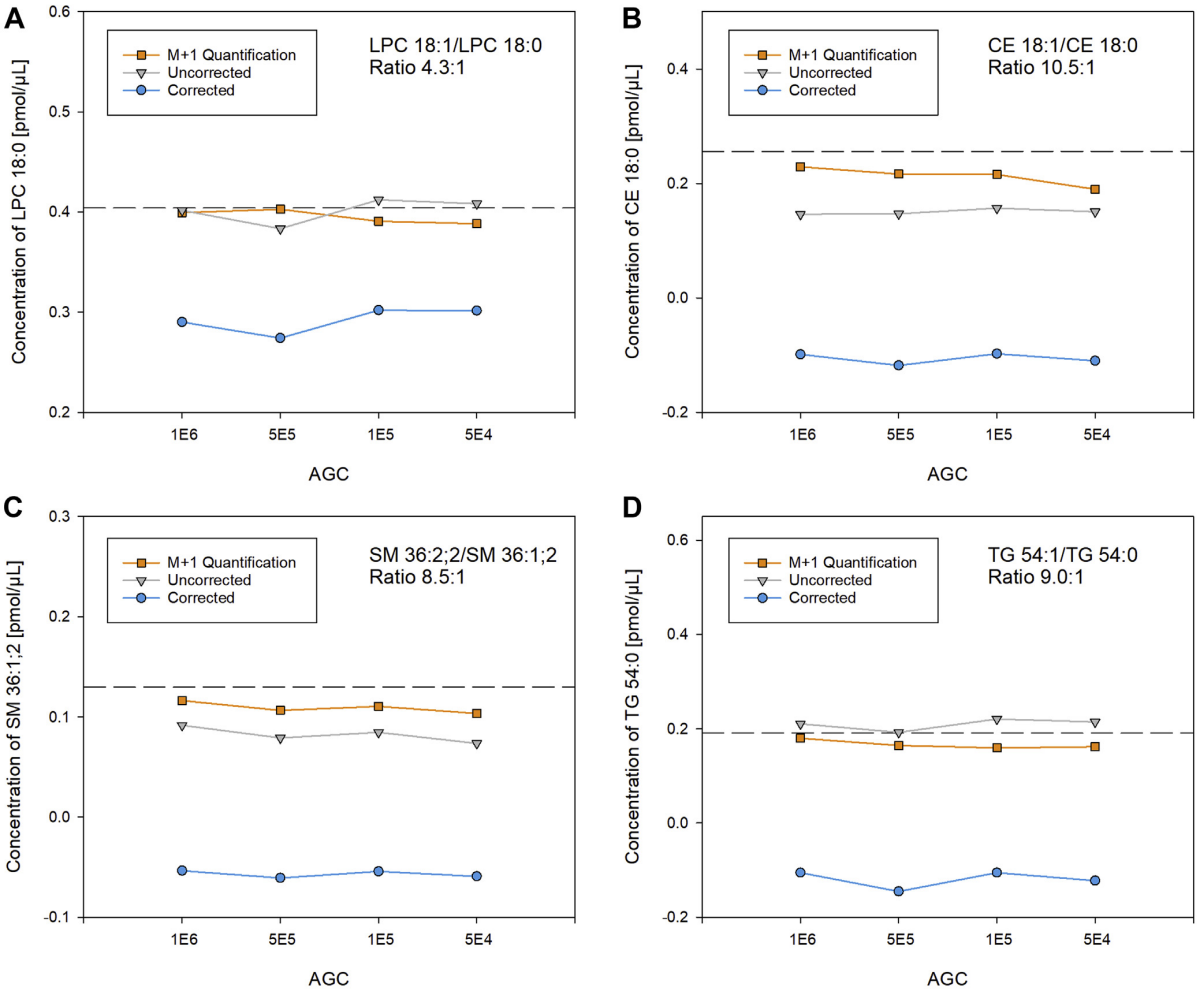
Effect of ion numbers (AGC levels). Quantification of (A) LPC 18:0 *m/z* 568.362, (B) CE 18:0 *m/z* 670.6497, (C) SM 36:1;2 *m/z* 775.5971 and (D) TG 54:0 *m/z* 908.8641 recorded at automated gain control (AGC) settings of 1 × 10^6^, 5 × 10^5^, 1 × 10^5^, and 5 × 10^4^ and resolution setting of 140,000 (at *m/z* 200). The dashed line indicates the spiked amount of the more saturated species. CE, cholesteryl ester; LPC, lysophosphatidylcholine; SM, sphingomyelin; TG, triglyceride.

Peak distortions in mass and intensity of ions with near-identical masses can be induced by peak coalescence, which strictly depends on the analyzed number of ions (14, 17). Hence, spike experiments were measured at different AGC settings. Lowering the number of ions from 1 × 10^6^ to 5 × 10^4^ did not affect the quantification for all compared correction algorithms (Fig. 5). Moreover, we did not observe any changes in the peak profiles (data not shown).

**Fig. 6:**
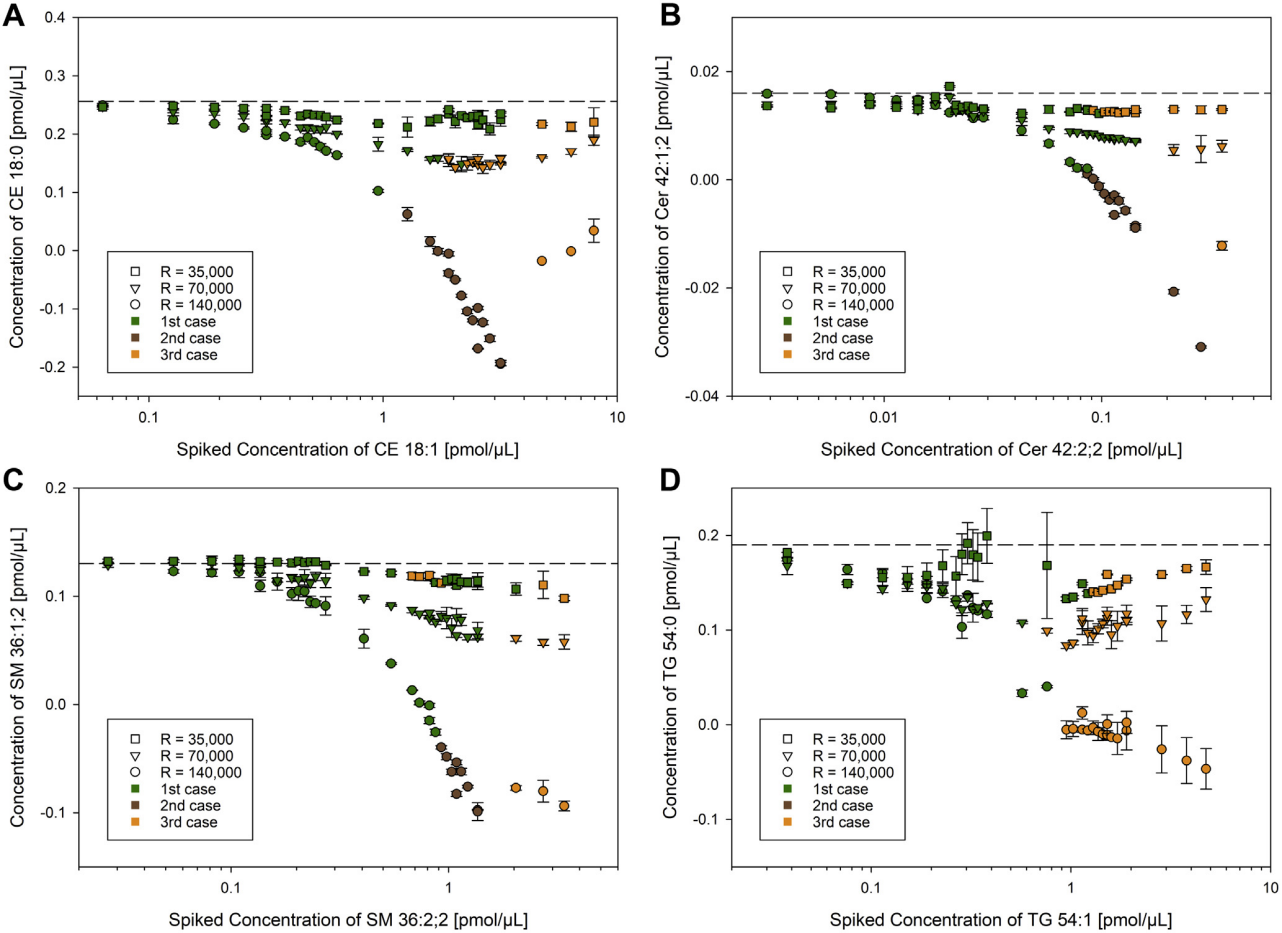
Reduction of the instrument resolution. Quantification of (A) CE 18:0 *m/z* 670.6497, (B) Cer 42:1;2 *m/z* 694.6355, (C) SM 36:1;2 *m/z* 775.5971, and (D) TG 54:0 *m/z* 908.8641 at increasing spike concentrations of the corresponding species with one additional double bond recorded on a QExactive at resolution settings of 140,000, 70,000, and 35,000 (*m/z* 200). All data were corrected for Type II effect (“corrected”). The dashed line indicates the target concentration of the saturated species. For third case peak configurations the intensity of M^i+1^+2 was picked at the apex and used for quantification of M^i^+0. Each point represents the average of n = 3 technical replicates ± SD. CE, cholesteryl ester; Cer, ceramide; SM, sphingomyelin; TG, triglyceride.

Theoretically, distortions induced by peak interference of ions with near-identical masses should decline by lowering the mass resolution resulting in additive intensities. Accordingly, the same samples were quantified at mass resolution settings of 70,000 and 35,000 (at *m/z* 200). Here, concentrations calculated after Type-II correction were more accurate at lower mass resolution arguing for a reduction of interference effects at lower resolutions (Fig. 6 and supplemental Fig. S2).

**Fig. 7:**
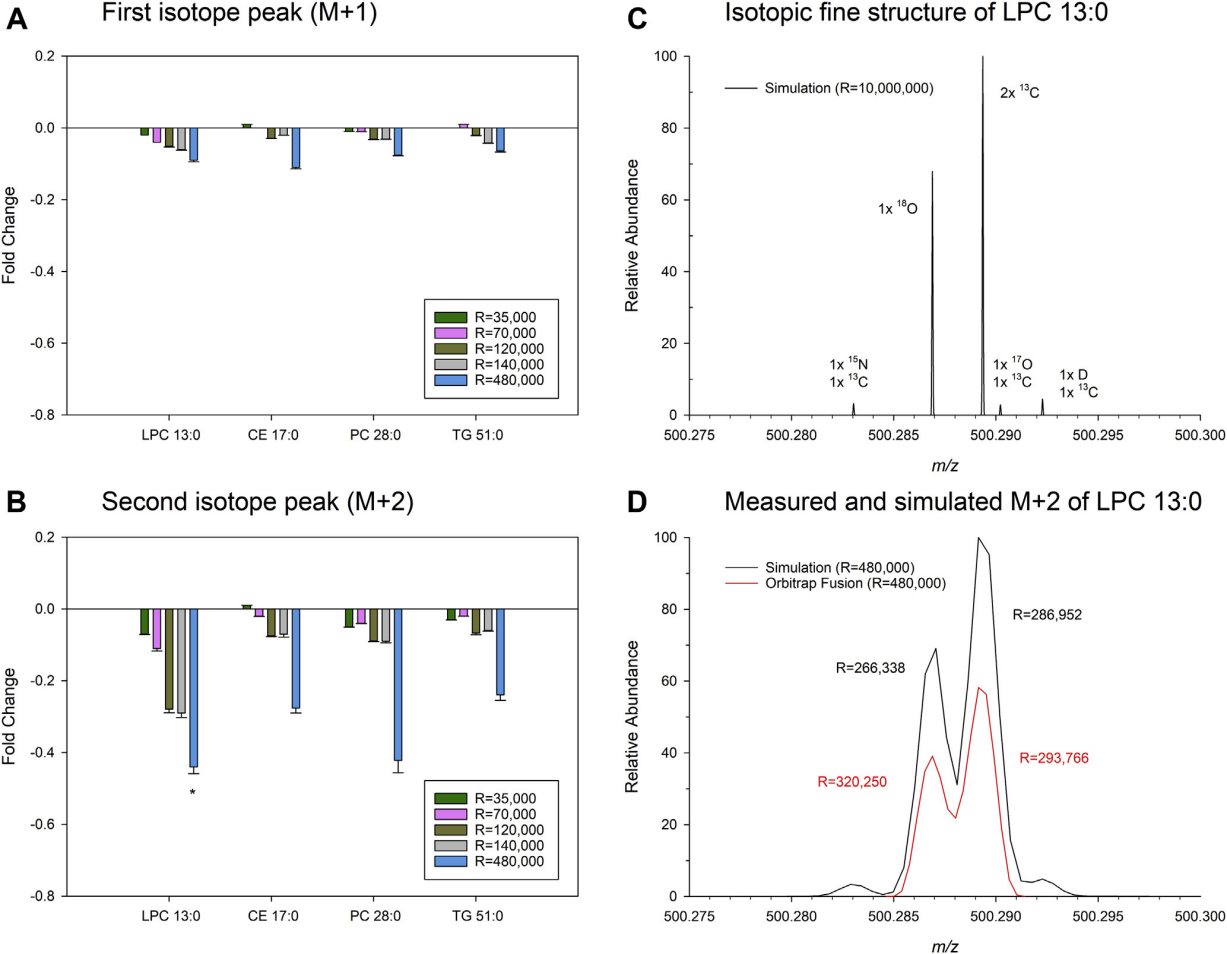
Relative isotopic abundance (RIA) at different resolution settings. Fold change (measured/calculated intensity) of the (A) first and (B) second isotope peak of LPC 13:0 (C_22_H_45_NO_9_P as [M+HCOO]^−^), PC 28:0 (C_36_H_73_NO_8_P as [M+H]^+^), CE 17:0 (C_44_H_82_NO_2_ as [M+NH_4_]^+^), and TG 51:0 (C_54_H_108_NO_6_ as [M+NH_4_]^+^). The isotopic fine structure of ^18^O and 2x^13^C was resolved at a resolution setting of 480,000. Therefore, the calculation of the second isotopic peak (M+2) of LPC 13:0 included only the peak containing 2x^13^C (∗). Each bar represents the average ± SD of n = 8 samples, each analyzed two times. Samples recorded at resolution settings of 35,000, 70,000, and 140,000 were analyzed on a QExactive Orbitrap and at resolutions of 120,000 and 480,000 on an Orbitrap Fusion. Panel (C) displays the simulation of the isotopic fine structure of LPC 13:0, and panel (D) shows the comparison of measured and simulated M+2 recorded at a resolution setting of 480,000 on an Orbitrap Fusion. CE, cholesteryl ester; LPC, lysophosphatidylcholine; PC, phosphatidylcholine; SM, sphingomyelin; TG, triglyceride.

Furthermore, we evaluated how mass resolution influences the RIA of internal standards, which were not affected by an overlap. LPC 13:0, CE 17:0, PC 28:0, and TG 51:0 were analyzed at different mass resolution settings with QExactive and Fusion Orbitrap instruments. Systematic trends were observed for the resolution dependent deviations in the calculated fractional abundance of M+1 and particularly M+2 (Fig. 7). The pronounced deviations of M+2 at 480,000 resolution could be explained by peak interference of the isotopic fine structure. In summary, these data demonstrate that peak interference is related to resolving power for both low and high-field instruments (9).

### Validation in biological specimen

Finally, the assay was evaluated and validated in biological specimen, i.e., human plasma and human skin fibroblasts. First, spike experiments could demonstrate linearity in a broad range for standard only as well as matrix containing samples (supplemental Figs. S3 and S4). A similar species response in the presence of matrix excludes significant effects of the matrix on species quantification.

Intraday and day-to-day precision were determined in human serum (supplemental Table S1) and skin fibroblasts (supplemental Table S2) for both uncorrected and M+1 quantification. For the majority of lipid species, coefficients of variation were below 10% with higher variations observed for M+1 for species with low abundance. Concentrations calculated from M+0 matched very well those derived from M+1 quantification with only a few exceptions for species with a concentration close to the limit of detection, e.g., DG 32:2 in fibroblasts. These species showed coefficients of variation of above 20% indicating that M+1-derived concentration is below limit of quantification (LOQ). Remarkably, minor species affected by substantial Type-II isotopic overlap like CE 18:0, TG 52:1 and PC 38:2 (in human plasma) were only accessible by M+1 quantification as, in contrast to the monoisotopic peak, the intensity of M+1 could be picked at the apex.

LOQ was determined as described before by fitting the determined accuracy and reproducibility for a concentrations series of spiked species (29, 31). For CE, DG, and PC LOQ was in the range of 2 nmol/ml and for TG at ∼0.5 nmol/ml (supplemental Table S3). For M+1, we expect accordingly an about 2- to 3-fold higher LOQ because of lower relative abundance of M+1 (30%–50% of monoistopic peak for these lipid classes).

## Discussion

In this study, we evaluated the quantification of lipid species by FIA-FTMS with particular consideration of the isobaric overlap occurring in double bond series. Peak distortions were observed when mass resolution was insufficient to resolve isobaric ions, which is in agreement with previous observations (9, 14, 15, 16, 17, 20). Because these distortions in mass and intensity were not affected by the number of collected ions peak coalescence as potential explanation could be excluded (14, 17). Instead, distortions seem to be related to the FTMS signal processing (16).

**Fig. 8:**
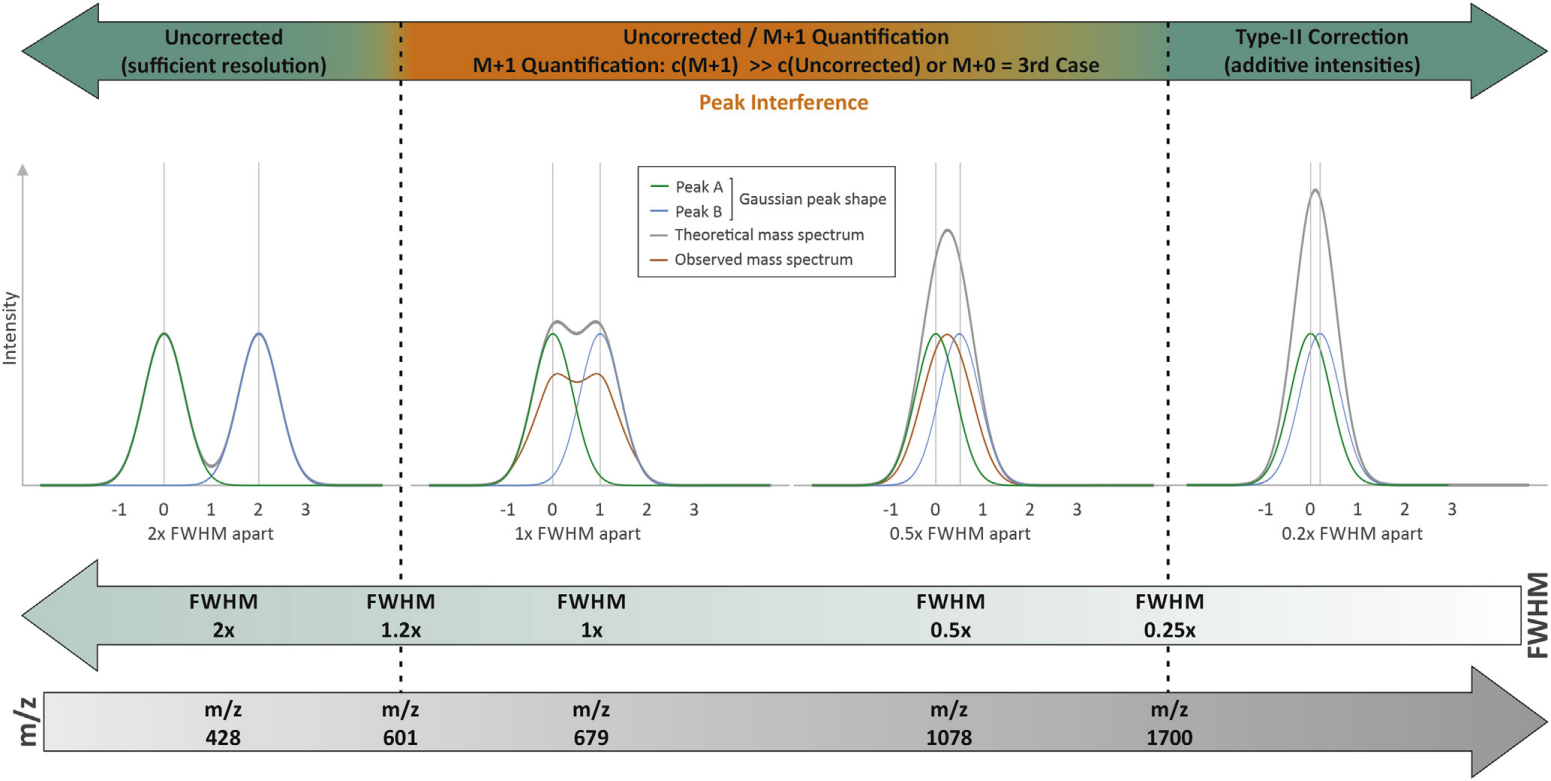
Consideration of Type-II isobaric overlap (8.94 mDa) in DB series. Displayed are equal abundant peaks (Gaussian peak shape model) resolved by 2×, 1×, 0.5×, 0.2× FWHM including their overlap mass spectra without (in gray) and with (in orange) peak interference. The suggested type of data correction is indicated for peak resolution/FWHM- and *m/z*–ranges that were calculated for a mass resolution setting of 140,000 (at *m/z* 200). DB, double bond; FWHM, FWHM, full-width at half maximum.

To develop rules for data processing, it is important to determine the *m*/*z* range, where peak interference occurs and how much it affects intensities for the investigated Type-II overlap. This could be inferred from partially resolved peaks and their deviations of the uncorrected from the M+1-derived as well as the target concentration. Furthermore, in case of completely unresolved peaks, the interference may lead to nonadditive intensities, which become evident when corrected are too low compared with target concentrations. Based on the data acquired at resolution setting of 140,000 (*m/z* 200), sufficient peak resolution and therefore no peak interference were observed for species at lower *m/z* (LPE 18:0 at *m/z* 478 and LPC 18:0 at *m/z* 568). More accurate M+1-derived concentrations were obtained for DG 36:0 at *m/z* 643; CE 18:0 at *m/z* 671; Cer 42:1;2 at *m/z* 695; SM 36:1;2 at *m/z* 776. For the Type-II overlap with mass difference of 8.94 mDa, a separation of equal abundant peaks by exactly 1× FWHM (Gaussian peak shape model) is achieved at approximately *m/z* 679. Based on our findings, peak interference should be considered starting at *m/z* 600 corresponding to a calculated peak resolution of about 0.8× FWHM (Fig. 8). Data acquired at lower mass resolutions settings were evaluated to estimate the upper end of peak interference induced distortions, where isobaric ions reveal additive intensities. In contrast to species at lower *m/z* (supplemental Fig. S2), Type-II corrected concentrations of CE 18:0 at *m/z* 670 and species at higher *m/z* (Fig. 6) showed acceptable accuracy compared with target concentrations at a resolution setting of 35,000 (at *m/z* 200). This corresponds to a theoretical peak separation of less than 0.25× FWHM. Adapted to a resolution setting of 140,000, the intensities of the isobaric ions should be additive for species of above *m/z* 1,700. However, further investigations are necessary to evaluate the range toward 0.25× FWHM peak separation. Importantly, beside the theoretical peak separation, the ratio of the isobaric ions needs consideration, which is in our workflow reflected by the peak configurations (Fig. 2). Thus, we recommend regarding only concentrations derived from first and second case (peak configurations) as reliable but not from third cases. This configuration-based concept applies for all presented types of quantification. Notably, the depicted color code represents the configuration of the monoisotopic peak. The configuration of M+1 is not visualized within the figures; however, third case configurations of M+1 were only observed for TG for the three samples with highest spike amounts of TG 54:1.

Considering accurate quantification, we recommend the calculation of both uncorrected and M+1-concentrations, in particular for respective *m/z*-ranges, where peak interference occurs (Fig. 8). Comparison of these concentrations does not only permit an easy quality check but also evaluation of peak interference effects. Moreover, some species affected by a substantial Type-II overlap could only be quantified using M+1 because peak interference is reduced because of substantially lower isotopic abundance of M^i+1^+3 (isobaric with M^i^+1) compared to M^i+1^+2. Therefore, M+1 quantification provides usually more accurate concentrations for such peaks. However, M+1 peak intensity is substantially lower compared with the monoisotopic peak compromising the sensitivity and thus analytical performance for minor species. Practically, we observed almost no deviations between uncorrected and M+1 concentrations for lipid species in plasma and fibroblast samples (supplemental Tables S1 and S2). Therefore, we suggest reporting of uncorrected concentrations, except M+1 shows a substantial increase compared to uncorrected concentration. In addition, M+1 concentrations should be reported when uncorrected values are derived from case 3; here, it is important to justify both that M+1 intensities are picked from the apex and the concentration exceeds the LOQ. More sophisticated decision rules could be developed based on a broader data basis and should therefore be subject to further studies. Furthermore, the excellent agreement of concentrations derived from M+0 and M+1 may be related to the good agreement of the RIA of M+1 (Fig. 7A). However, we observed a clear trend to underestimate the isotopic peaks with increasing mass resolution. Thus, M+1-derived concentrations may be underestimated by about 5% at a resolution setting of 140,000 (*m/z* 200).

Alternative approaches to circumvent the described peak interference include application of higher mass resolutions (e.g., 480,000 at *m/z* 200), which can be achieved either by advanced instrumentation (9), acquisition of longer transients (17), or alternative signal processing methods like the recently published phased spectrum deconvolution method (ΦSDM) (32).

In summary, we could show that lipidomic data acquired by Orbitrap FTMS may suffer from peak interference caused by the Type-II overlap in DB series. The proposed strategy to use M+0 and M+1 for quantification could be applied to identify isobaric peak interference as well as to improve accuracy of quantification and species coverage. Method validation could show sufficient linearity, a negligible effect of sample matrix on the species response and a high reproducibility of the quantification (including M+1). Further isobaric interferences may derive from other adduction ions like sodium requiring additional correction (33). Accurate lipid species quantification is not only key for successful lipidomic research (34) including clinical studies (35) but also relates to the major aims of the recently founded Lipidomics Standards Initiative (https://lipidomics-standards-initiative.org/) (36).

## Data availability

The data supporting this study are available in the article, the supplemental data, or available from the corresponding author upon reasonable request.

## Supplemental data

This article contains supplemental data.

## Conflict of interest

The authors declare that they have no conflicts of interest with the contents of this article.
