# Supplementary material for: Accurate quantification of lipid species affected by isobaric overlap in Fourier-transform mass spectrometry
Source: J Lipid Res. 2021 Feb 16;62:100050. doi: 10.1016/j.jlr.2021.100050 (PMC8010702; doi:10.1016/j.jlr.2021.100050)
Supplement: Supplemental Figs. S1 to S4 [file mmc1.pdf]

SUPPLEMENTAL INFORMATION

Accurate quantification of lipid species affected by isobaric overlap in Fourier-Transform mass spectrometry

Marcus Höring[1], Christer S. Ejsing[2,3], Sabrina Krautbauer[1], Verena M. Ertl[1], Ralph Burkhardt[1], Gerhard Liebisch[1,*]

1) Institute of Clinical Chemistry and Laboratory Medicine, Regensburg University Hospital, 93053 Regensburg, Germany

2) Department of Biochemistry and Molecular Biology, Villum Center for Bioanalytical Sciences, University of Southern Denmark, 5230 Odense, Denmark

3) Cell Biology and Biophysics Unit, European Molecular Biology Laboratory, 69117 Heidelberg, Germany

Table of Content

Figure S1. .... S2

Figure S2. .... S3

Figure S3. .... S4

Figure S4. .... S5

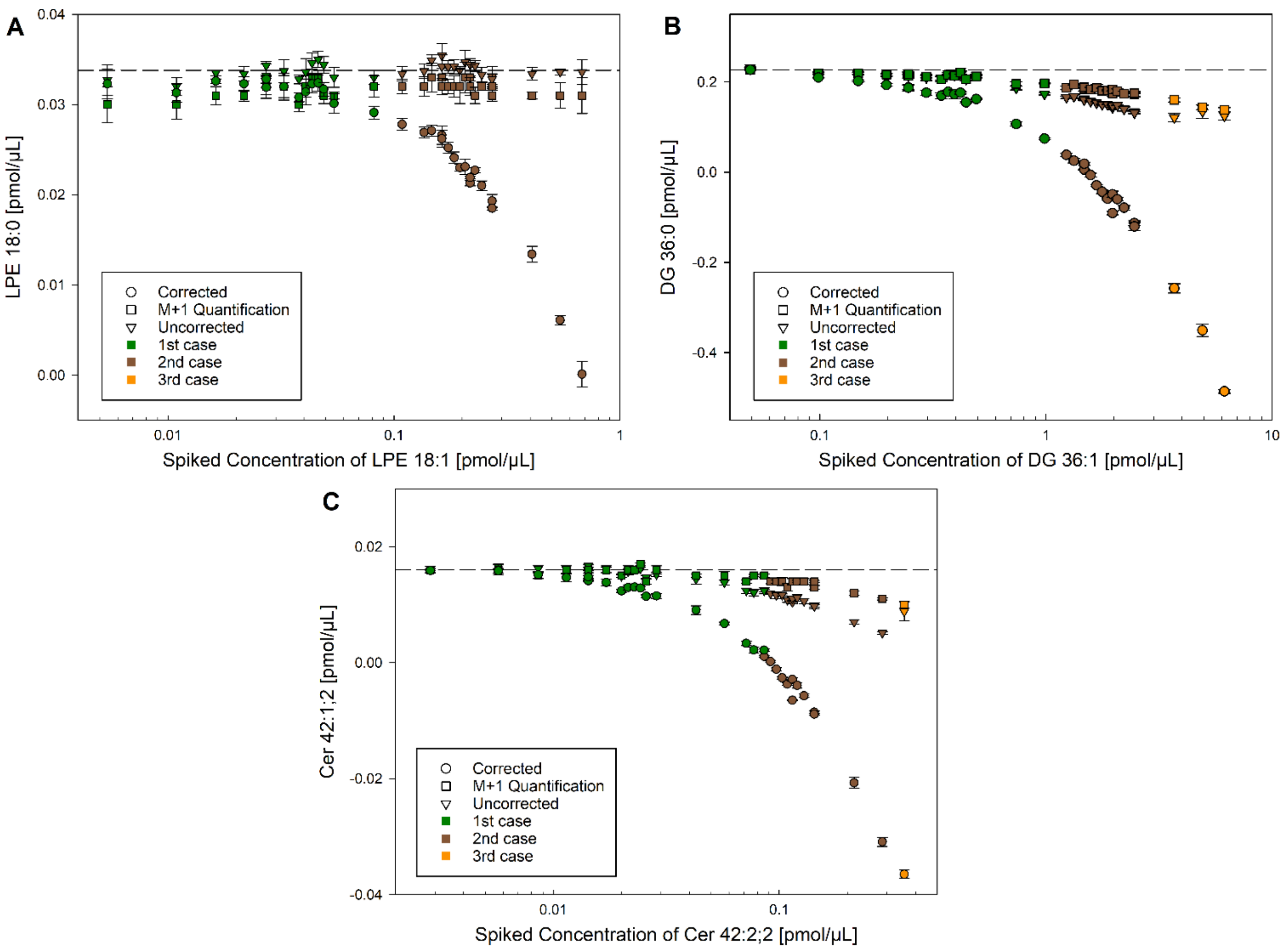

Figure S1: Comparison of data processing strategies. Quantification of (A) LPE 18:0 *m/z* 480.3096, (B) DG 36:0 *m/z* 642.6031, and (C) Cer 42:1;2 *m/z* 694.6355 at increasing concentrations of the corresponding species with one additional double bond at resolution setting of 140,000 (at *m/z* 200). The figure displays concentrations calculated without (uncorrected, triangles), with (corrected, circles) Type-II correction and by the use of M+1 (squares). The dashed line indicates the spiked amount of the more saturated species. The color code describes the case of peak configuration (M^{i+1}+2 and M^{i}+0). Each point represents the average of n = 3 technical replicates ± SD.

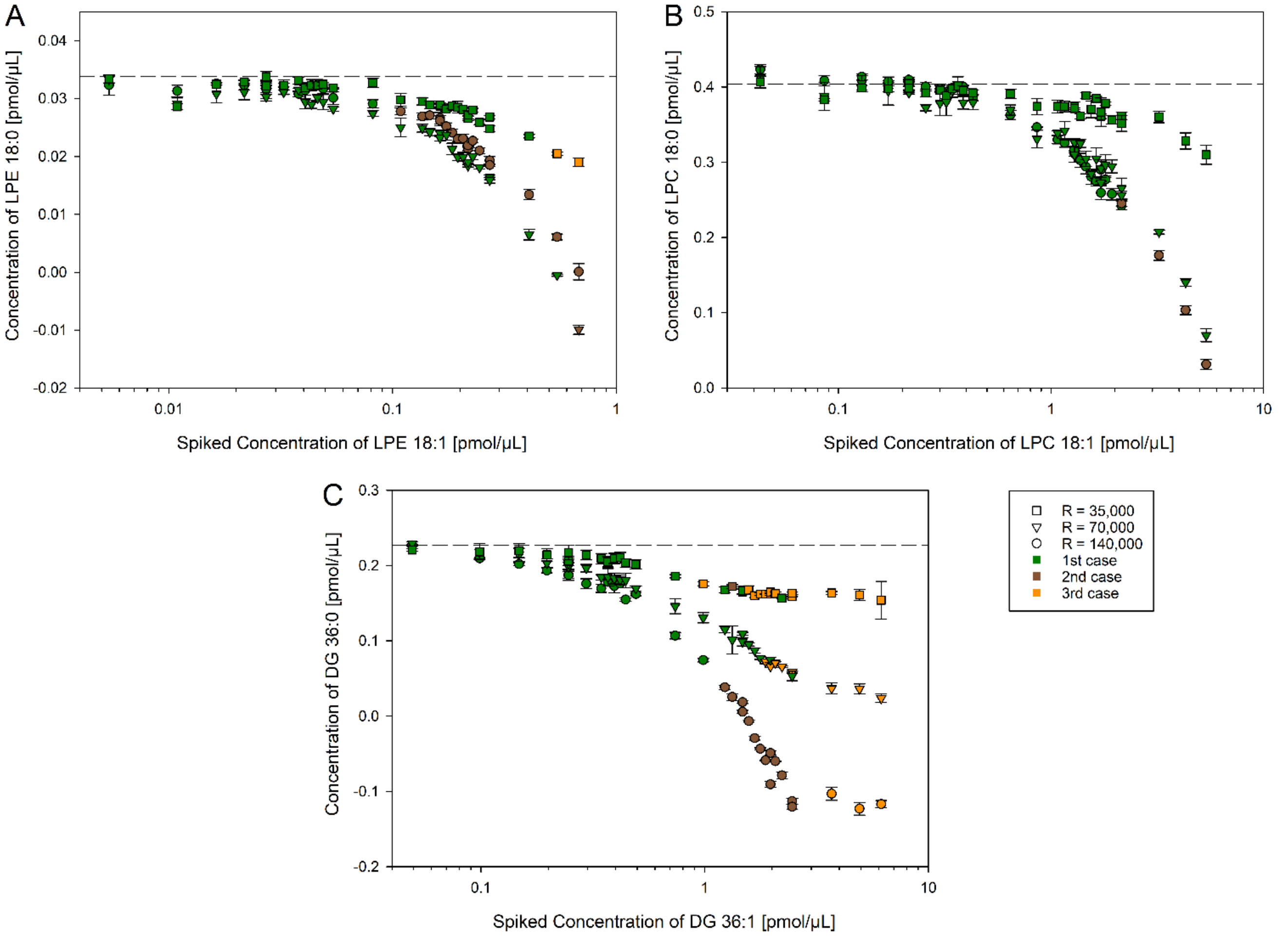

Figure S2: Reduction of the instrument resolution. Quantification of (A) LPE 18:0 *m/z* 480.3096, (B) LPC 18:0 *m/z* 568.362, and (C) DG 36:0 *m/z* 642.6031 at increasing spike concentrations of the corresponding species with one additional double bond recorded on a QExactive at resolution settings of 140,000, 70,000, and 35,000 (*m/z* 200). All data were corrected for Type II effect ("corrected"). The dashed line indicates the target concentration of the saturated species. For third case peak configurations the intensity of $M^{i+1}+2$ was picked at the apex and used for quantification of $M^{i}+0$. Each point represents the average of n = 3 technical replicates ± SD.

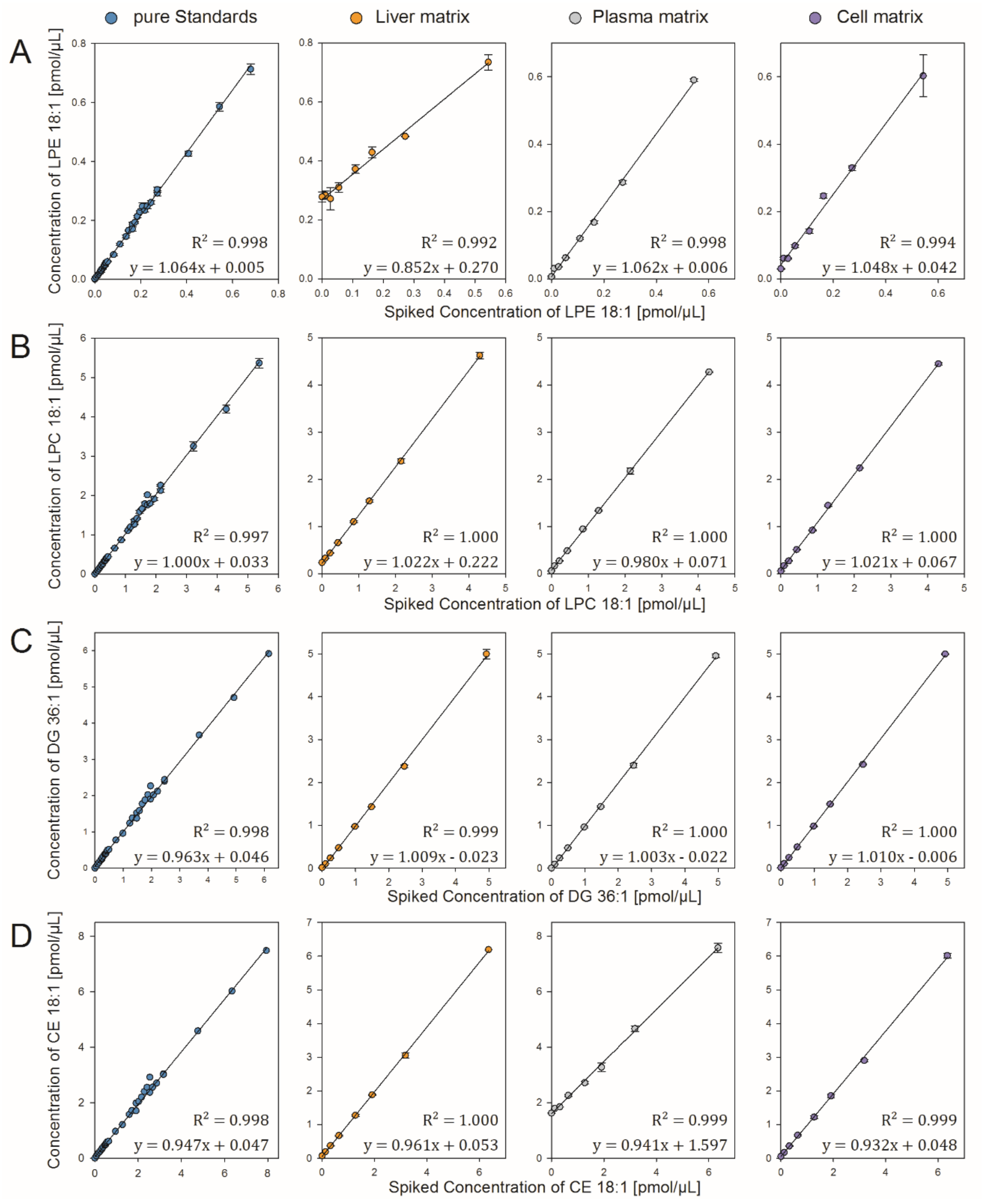

Figure S3: Dynamic range of quantification. FTMS analysis of (A) LPE 18:1, (B) LPC 18:1, (C) DG 36:1, and (D) CE 18:1 using a target resolution of 140,000 at *m/z* 200. Each synthetic standard was analyzed (from left to right) without matrix and spiked on murine liver, human plasma and human fibroblast cells. The figures display the correlation of measured and spiked concentration. Each data point represents the average of three replicate analyses per sample.

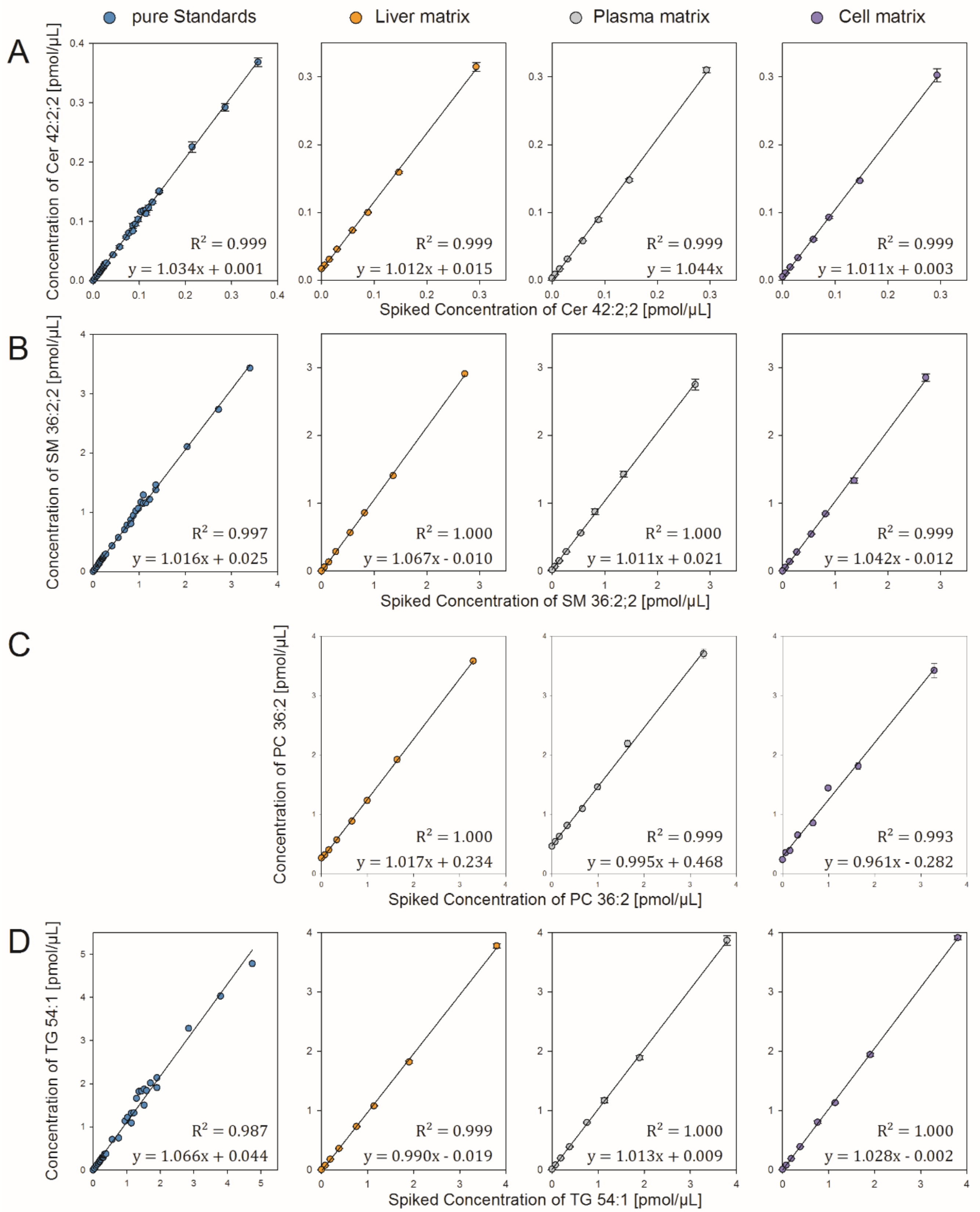

Figure S4. Dynamic range of quantification. FTMS analysis of (A) Cer 42:2;2, (B) SM 36:2;2, (C) PC 36:2, and (D) TG 54:1 using a target resolution of 140,000 at *m/z* 200. Each synthetic standard was analyzed (from left to right) without matrix (not performed for PC 36:2) and spiked on murine liver, human plasma and human fibroblast cells. The figures display the correlation of measured and spiked concentration. Each data point represents the average of three replicate analyses per sample.
